# On the Chemical Changes of Respiration

**Published:** 1849-06

**Authors:** 


*On the Chemical Changes of Respiration.* By MM. REGNAULT and REISET.—A new and very extended series of experiments on this subject have been instituted by MM. Regnault and Reiset, who give minute details of the several steps of the process employed by them, the precautions taken, and the kind of apparatus used. Their investigations, which are still in progress, seemed to be performed with much care and exactness, and their results may probably be fully relied on. The most important of these results is, that nitrogen is invariably exhaled through the lungs, though the quantity is small, rarely exceeding one one-hundredth of the amount of oxygen consumed. Hydrogen, and certain carburetted gases, usually present themselves in small quantity. As an illustration of the changes which Regnault and Reiset found to occur in the respired air, the following results of an experiment, in which a young dog was confined in the apparatus for twenty-four hours and a half, may be quoted:—

| | Grammes. |
|---|---|
| Oxygen consumed | 182.288 |
| Carbonic acid produced | 185.961 |
| Oxygen contained in the carbonic acid | 135.244 |
| Nitrogen disengaged | 0.1820 |

If the quantity of oxygen consumed be represented at 100, then the results may be thus stated:—

| | |
|---|---|
| Oxygen consumed | 100 |
| Oxygen in the carbonic acid | 74.191 |
| Oxygen otherwise disposed of | 25.809 |
| Nitrogen disengaged | 0.0549 |
| Average quantity of oxygen consumed in an hour | 7.44 |

These experiments rectify the erroneous results of Dulong and Despretz, who found that the quantity of nitrogen disengaged during respiration, was sometimes as great as one-fourth the quantity of oxygen absorbed.—*London Monthly Journal, from Comptes Rendus.*

